# The Lamellar Cells of Vertebrate Meissner and Pacinian Corpuscles: Development, Characterization, and Functions

**DOI:** 10.3389/fnins.2022.790130

**Published:** 2022-03-09

**Authors:** Iván Suazo, José A. Vega, Yolanda García-Mesa, Jorge García-Piqueras, Olivia García-Suárez, Teresa Cobo

**Affiliations:** ^1^Grupo SINPOS, Departamento de Morfología y Biología Celular, Universidad de Oviedo, Oviedo, Spain; ^2^Faculcultad de Ciencias de la Salud, Universidad Autónoma de Chile, Santiago, Chile; ^3^Departamento de Cirugía y Especialidades Médico-Quirúrgicas, Universidad de Oviedo, Oviedo, Spain

**Keywords:** lamellar cells, sensory corpuscles, mechanoreceptors, ion channels, mechanotransduction

## Abstract

Sensory corpuscles, or cutaneous end-organ complexes, are complex structures localized at the periphery of Aβ-axon terminals from primary sensory neurons that primarily work as low-threshold mechanoreceptors. Structurally, they consist, in addition to the axons, of non-myelinating Schwann-like cells (terminal glial cells) and endoneurial- and perineurial-related cells. The terminal glial cells are the so-called lamellar cells in Meissner and Pacinian corpuscles. Lamellar cells are variably arranged in sensory corpuscles as a “coin stack” in the Meissner corpuscles or as an “onion bulb” in the Pacinian ones. Nevertheless, the origin and protein profile of the lamellar cells in both morphotypes of sensory corpuscles is quite similar, although it differs in the expression of mechano-gated ion channels as well as in the composition of the extracellular matrix between the cells. The lamellar cells have been regarded as supportive cells playing a passive role in the process of genesis of the action potential, i.e., the mechanotransduction process. However, they express ion channels related to the mechano–electric transduction and show a synapse-like mechanism that suggest neurotransmission at the genesis of the electrical action potential. This review updates the current knowledge about the embryonic origin, development modifications, spatial arrangement, ultrastructural characteristics, and protein profile of the lamellar cells of cutaneous end-organ complexes focusing on Meissner and Pacinian morphotypes.

## Introduction

A subset of peripheral axon terminals from primary sensory nerve fibers reaches the vertebrate dermis and contacts with distinct differentiated cells to form microscopic sensory organs referred to as sensory corpuscles (Zimmerman et al., 2014; Cobo et al., 2021). Recently, Handler and Ginty (2021) used the term cutaneous end-organ complexes (CEOCs) to refer to these structures, which is surely more appropriate. CEOCs are continuous with nerve fibers whose axons originate from intermediate- or large-sized mechanosensory neurons that work as low threshold mechanoreceptors (LTMRs) [see for a review (Abraira and Ginty, 2013)]. LTMRs can be divided into two categories: rapidly adapting (RA) and slowly adapting (SA) mechanoreceptors. Both, in turn, have type I and type II variants (Zimmerman et al., 2014; Olson et al., 2016; Cobo et al., 2021). RA type I are Meissner corpuscles, RA type II correspond to Pacinian corpuscles, SA type I are the Merkel cell–neurite complexes, and SA type II are the dermal Ruffini’s corpuscles. They are widely distributed throughout the body but are especially abundant in the skin where there is high sensibility to touch and transduce different qualities of mechanosensitivity (Zimmerman et al., 2014; Cobo et al., 2021).

The cutaneous cells contacting the axon terminal of LTMRs, especially the Aβ ones (Abraira and Ginty, 2013), are specialized epithelial cells or glial Schwann-like cells: the axon–epithelial cell associations form the Merkel cell–neurite complexes or touch discs; the sets of axon and glial Schwann-like cells (terminal glial cells) form the core of CEOCs, i.e., Meissner corpuscles, Ruffini’s corpuscles, and Pacinian corpuscles (Munger and Ide, 1988; Zelená, 1994; Zimmerman et al., 2014; Cobo et al., 2021). Therefore, CEOC structurally consists of the axon terminal (also called dendritic zone) of one LTMR and non-myelinating terminal glial cells variably arranged depending on the morphotype of the corpuscle, and both are surrounded by a capsule of endoneurial/perineurial cells (Vega et al., 1996, 2009; García-Piqueras et al., 2017, 2020a; Cobo et al., 2021). So, the periaxonal cells within sensory corpuscles are continuous with the periaxonal cells on the nerve fibers [see (Cobo et al., 2021)].

The glial cells forming a part of CEOCs are a special subpopulation of peripheral glial cells named terminal glial cells or skin end-organ glia (Kastriti and Adameyko, 2017). These cells are habitually neglected in neurohistology books and reviews [see (Reed et al., 2021)] and were classically regarded as supportive and inert, and passive in the genesis of the action potential since they are non-excitable cells. However, emerging data support that terminal glial cells of CEOCs are fundamental in the process of mechanotransduction, but the putative role of the terminal glia on somatosensation remains largely unknown.

Most of the data collected for this review come from humans, although other vertebrates, especially mice, are also mentioned. To avoid confusion about which cells are reviewed here, it must be clarified that laminar cells are understood, exclusively, as the terminal glial cells, which form the lamellae arranged between or around the axon terminal. In some morphotypes of CEOCs there are other cells that also adopt a laminar morphology or arrangement but are not glial cells, for example, those that form the intermediate layer, the outer core, and the capsule in Pacinian corpuscles, or the capsule in Meissner corpuscles, but those cells are not the objective of this review. Here, we review and update the embryonic origin, development, morphology, ultrastructure, immunohistochemical profile, and putative functions of the terminal glial cells within the CEOCs, especially Meissner and Pacinian corpuscles.

## Meissner Corpuscles

**FIGURE 1:**
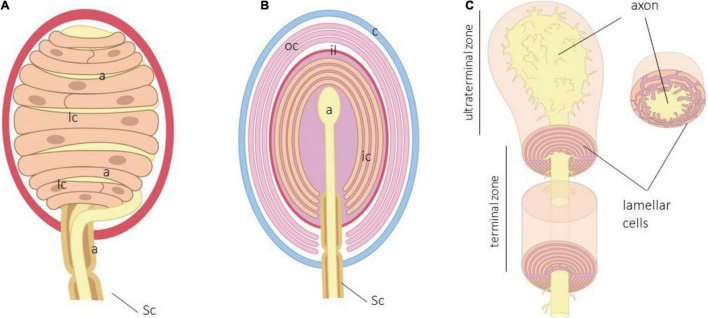
**(A)** Schematic representation of human digital Meisser. a, axon; c, capsule; lc, lamellar cells, Sc, Schwann cells. Lamellar cells in Meissner corpuscles are closely related to the axon and form a “coin stack.” **(B)** Schematic representation of human Pacinian corpuscle. a, axon; c, capsule; ic, inner core; il, intermediate layer; lc, lamellar cells; oc, outer core; Sc, Schwann cells. **(C)** Arrangement of the terminal glial cells in the terminal and ultraterminal zones of the inner core.

Meissner corpuscles (Figure 1A) are exclusive in humans and primates, although similar structures, i.e., Meissner-like corpuscles, are also present in other mammals (Zelená, 1994). They are typical of the glabrous skin but concentrate in areas of fine touch discriminative capacities (fingertips, palm, sole of the feet, lips, and male and female genital skin) and occasionally on the tongue and palate (Vega et al., 1996, 2009).

Meissner corpuscles are found in the dermal papillae, just below the basal lamina of the epidermis. They have a typical oval shape with the major axis perpendicular to the surface of the skin, but its morphology and size are largely variable. Structurally, they consist of one single axon, terminal glial cells, and a capsule. In humans, the axon is usually unique, whereas in *Macaca fascicularis* and *Macaca mulata*, multiple axons can be detected, the main one being of Aβ type, and the accessories are the ones of C or Aδ type (Paré et al., 2001). Very recently, Neubarth and coworkers (Neubarth et al., 2020) observed that murine Meissner-like corpuscles contain two mechanoreceptor axon subtypes with distinct responses to tactile stimuli. The myelin sheath that envelops the axon of the nerve fiber supplying the corpuscle is lost upon entering the corpuscle (García-Suárez et al., 2009), and the axon is coated by Schwann-related cell-denominated laminar cells. They are arranged as stacks of flattened sheets (classically described as in a “coin stack”) parallel to the skin surface. Typically, the lamellar cells possess a basal lamina (Vega et al., 1995), and the interlamellar space is filled with a chemically complex extracellular matrix. Delimiting the corpuscle, adapted at the external side of the lamellar cells, there is a capsule of CD34 + positive endoneurial cells (García-Piqueras et al., 2020a).

Functionally, Meissner’s corpuscles are RA type I-LTMRs that detect fine touch, vibration, and fine movements on the skin (Johnson et al., 2000). Furthermore, Meissner’s corpuscles in monkeys could also work as nociceptors since accessory axons contain nociceptive neuropeptides (Paré et al., 2001).

## Pacinian Corpuscles

Pacini’s corpuscles (Figure 1B) are large ovoid formations distributed in most organs and tissues, including the deep dermis and hypodermis (Bell et al., 1994; Zelená, 1994). Cells forming Pacinian corpuscles are typically arranged forming hemilamellae and lamellae around the axon to show a typical appearance of an “onion bulb.”

In the central part of the corpuscle is the axon of an Aβ mechanosensory neuron. The periaxonal cells of Pacinian corpuscles form three distinct compartments denominated as the inner core, outer core, and capsule. The inner core consists of hemilamellae of non-myelinating terminal glial cells, while the outer core and the capsule are composed of concentrically arranged flattened lamellae of perineurial fibroblast-like cells; the interlamellar spaces of the capsule typically contains capillaries and macrophages. Between the inner and the outer core, there is an intermediate cell stratum, called intermediate layer of growth layer, whose cellular elements are modified endoneurial CD34 + fibroblasts (García-Piqueras et al., 2017). Among the lamellae of the inner core, outer core, and capsule, there is a fluid-filled cellular space containing a molecularly complex extracellular matrix, including collagen (Vega et al., 1995; Dubový and Bednárová, 1999; Sames et al., 2001; García-Piqueras et al., 2019a,b, 2020b).

Functionally, Pacini’s corpuscles are RA type II-LTMRs and respond to pressure and vibratory stimuli between 20 and 1,500 Hz, with a maximum sensitivity at 200–400 Hz (Jones and Smith, 2014; Olson et al., 2016).

## The Lamellar Cells of the Meissner and Pacinian Corpuscles

### Cytoarchitecture and Ultrastructure

The spatial arrangement of the cells forming sensory corpuscles, i.e., axon, terminal glial cells, endoneurial-, and perineurial-like cells, varies according to the morphotype of the CEOCs. In particular, the terminal glial cells can be disposed within the corpuscle irregularly (Krause and Ruffini corpuscles), parallel (Meissner corpuscles), or concentric (Pacinian corpuscles).

#### Meissner Corpuscles

The terminal glial cells of Meissner corpuscles are usually denominated lamellar cells because their typical horizontal flattened appearance that form stacks of lamellae (“coin stack”) between the lamellar cells are the axon branches (Munger and Ide, 1988; Vega et al., 2012). The lamellae are divided by relatively wide interspaces that contain the extracellular matrix components, and septal partitions may subdivide the corpuscle into two or more compartments. The lamellar cell bodies, containing the nuclei, are localized at the periphery of the corpuscle and stretch their flat cytoplasmic processes across. Their lamellae contain only a few mitochondria and other organelles but are rich in pinocytotic vesicles related to the mechanism of endocytosis/exocytosis (Munger and Ide, 1988; Zelená, 1994).

In murine digital Meisser-like corpuscles, using freeze–fracture techniques, it has been observed that the membranes of the axon terminals and lamellar cells contain intramembranous particles about 10 nm in diameter. The density of these particles in the axon membranes was somewhat lower than that of the lamellar cell membranes, and presumably, they have specific physiological properties in mechanoreceptive functions including mechano–electric transduction [see (Ide et al., 1985; Munger and Ide, 1988)].

#### Pacinian Corpuscles: The Inner Core

The terminal glial cells of Pacinian corpuscles form the inner core, which lies between the axon and the intermediate layer. The inner core starts where the myelin ends, and it consists of tightly packed lamellae that originate from the lamellar cells whose bodies are disposed at the periphery of the core.

Within the inner core, the lamellae are diversely arranged in the preterminal, terminal, and ultraterminal zones of the corpuscle (Bell et al., 1994; Zelená, 1994). In the *preterminal zone*, they fully encircle the initial intracorpuscular segment of the terminal axon and are myelinating cells. In the *terminal zone*, the lamellae adopt a bilateral symmetric organization: the inner core is formed by two bilaterally symmetric hemilamellae systems in which the ends are opposite each other that corresponds to the longer axis of the axon. So, in the terminal zone, the inner core, the hemilamellae, forms dual gaps of radial clefts set 180° apart; processes of the axon terminal, called axonal spines, protrude into the radial clefts. Finally, the lamellae of the *ultraterminal zone* loses bilaterally, and small portions of their cytoplasm are disposed irregularly to surround the bulbous terminal of the axon (Figure 1C).

The lamellae of the inner core are very thin and contain mitochondria, concentric arrays of rough endoplasmic reticulum, and other membranous structures, as well as pinocytotic vesicles. The lamellar tips that abut the axon terminal are sometimes filled with dense cored vesicles and tubules. On the other hand, the lamellae are connected with many tight junctions. They are further joined with numerous gap junctions, which could allow a free flow of ions between individual inner core cells. Attachment plaques characterized by dense undercoating frequently connect successive lamellae and lamellar tips, but true desmosomes have been, so far, not encountered in freeze–fracture replicas. These attachment plaques probably ensure mechanical cell-to-cell attachment and, thus, contribute to the mechanical stability of the inner core together with tight junctions [see for a review (Munger and Ide, 1988; Bell et al., 1994; Zelená, 1994)].

## Origin and Development of the Lamellar Cells

The elegant studies carried out by review in Saxod (1978, 1996) demonstrated that the development of cutaneous sensory corpuscles results from complex morphogenetic interactions between the dermal mesenchyme, the somatosensory nerve, and neural crest-derived cells. These cells, considered originally as specialized Schwann cells (Idé, 1977), are the origin of the lamellar cells in the cutaneous sensory corpuscles in higher vertebrates, including humans.

**FIGURE 2:**
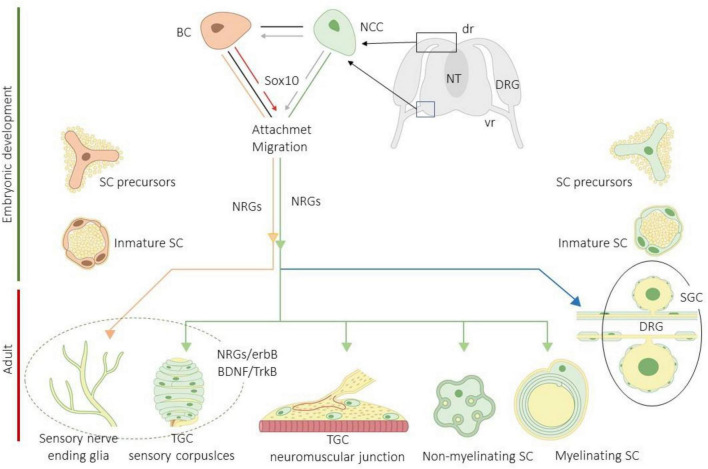
Schematic representation of development and differentiation of peripheral glial cells subtypes. BC, boundary cells; BDNF, brain-derived neurotrophic factor; DRG, dorsal root ganglia (dr, dorsa root; vr, ventral root); erbBs, neuregulin receptors; NCCs, neural crest cells; NRGs, neuregulins; NT, neural tube; SCs, Schwann cells; SGCs, satellite glial cells; TGCs, terminal glial cells; TrkB, high affinity receptor for BDNF. Imitated from Kastriti and Adameyko (2017).

The progenitors of the peripheral glial cells originate mainly from neural crest cells (NCCs) or from cap boundary cells (BCs). These cells contact and attach to the surface of developing axons and differentiate into Schwann cell precursors (S) and then into immature Schwann cells (ISCs) under the action of different molecules, especially neuregulins (NRGs). The ISCs give rise to a pleiad of peripheral glia including myelinating and non-myelinating Schwann cells, the satellite glial cells of the peripheral sensory and vegetative ganglia, the enteric glia, the terminal glial cells of the neuromuscular junctions and CEOCs (also denominated end-organ glia), and the sensory nerve fiber-associated glia (SNF-AG). Most of those cells originate directly from NCCs, and only the glial cells associated with cutaneous sensory nerves derivate from BCs (Maro et al., 2004; Aquino et al., 2006; Kastriti and Adameyko, 2017; Petersen and Adameyko, 2017; Furlan and Adameyko, 2018; Figure 2).

Neural crest cells and boundary cells migrating toward the skin attach the growing sensory axons together with other nerve-associated cells, especially fibroblasts, and in the dermis, they give rise to highly specialized glial cells denominated SNF-AGs, which are associated to intraepidermal nerve endings (i.e., the adult intraepidermal free nerve endings). Nevertheless, SNF-AGs are different from the terminal glial cells of the sensory corpuscles since, whereas SNF-AGs are in contact with C-o Aδ fibers, the terminal glial cells are in relation to Aβ fibers (Gresset et al., 2015). Therefore, NCCs and BCs originate from at least three cutaneous glial populations: one type of Schwann cells (mainly non-myelinating) in relation with subcutaneous and dermal nerves and the CEOCs connected to them, and two types of terminal Schwann cells associated with lanceolate endings (in the hairy skin) and free nerve endings (in the glabrous and hairy skin). At present, the precise origin of the terminal glial cells within sensory corpuscles is still unknown, but probably, they derivate from NCCs (Etxaniz et al., 2014). The final phenotype of the cutaneous glial cells is probably a consequence of both specific molecular and local factors (Kastriti and Adameyko, 2017; Jessen and Mirsky, 2019). Interestingly, some SCPs remain undifferentiated postnatally in the dermis and peripheral nerves, and retain embryonic properties, that is, they do not completely lose multipotentiality (Widera et al., 2011; Calavia et al., 2012; Dyachuk et al., 2014).

Sensory axons are critical for the development of cutaneous CEOCs. Bidirectional interactions between sensory axons and SCPs, mediated by NRGs, initiate their morphogenesis (Saxod, 1996). In fact, the interaction of SCPs with growing axons (Jessen and Mirsky, 2005; Nave and Salzer, 2006) through NRG-1/ErbB2/ErbB3 receptors induce the comigration (Birchmeier and Nave, 2008), and a RET-ER81-NRG-1 signaling pathway promotes reciprocal interactions between axon and non-myelinating Schwann cells (Fleming et al., 2016). The terminal glial cells forming the inner core of murine Pacinian corpuscles display erbB2, erbB3, and erbB4 NRG receptors, whereas the central axon contains NRG-1, and deletions of *Ret* and *Nrg1* in mechanosensory neurons results in the absence of Pacinian corpuscles, while Meissner corpuscles are unaffected (González-Martínez et al., 2007; Luo et al., 2009). Conversely, the functional complex of brain-derived neurotrophic factor and TrkB (BDNF/TrkB), control the development of Meissner corpuscles without effect on Pacinian corpuscles indicating a role in the development of specific mechanosensory neurons (LeMaster et al., 1999; Ichikawa et al., 2000; González-Martínez et al., 2004, 2005; Krimm et al., 2006; Pérez-Piñera et al., 2008). Nevertheless, other members of the NT family could also participate in the development of Pacinian corpuscles (Sedý et al., 2004). On the other hand, ER81, a member of the E26 transformation-specific family of transcription factors, is expressed in the inner core cells of Pacinian corpuscles, and the *Er81*-null mice lack of Pacini corpuscles is presumably because the sensory neurons subserving Pacinian corpuscles do not survive (Sedý et al., 2006).

In addition to the molecules that are involved int the axon–glial cells interactions, or those that control indirectly the CEOC development by regulating neuronal survival, local molecules also seems to be involved in their development. Among them are target-derived growth factors (Schecterson and Bothwell, 1992; Dontchev and Letourneau, 2003), β-arrestin-1 (Komori et al., 2003), semaphorins (Dontchev and Letourneau, 2003; Curley et al., 2014; McCormick et al., 2015), ankyrin-B (Engelhardt et al., 2013), and even mechanical signals (Koser et al., 2016).

To study the temporal pattern of CEOC development, specific markers for the different constituents (axon, terminal cells, endoneurial, and perineurial cells) are currently used in immunohistochemical studies. Interestingly, these studies are facilitated by the fact that NCCs, SCPs, ISCs, and mature Schwann cells have common markers, including nestin, vimentin, and S100β protein (Jessen and Mirsky, 2005; Engelhardt et al., 2013). Using antibodies against these proteins, we established the timetable of the development of human digital CEOCs (Feito et al., 2018).

### Meissner’s Corpuscles

Meissner’s corpuscles, which start differentiation at around 20 weeks of estimated gestational age (WEGA), show basic morphology by 36 WEGA, and acquire the definitive aspect and immunohistochemical profile postnatally. Nevertheless, the density of Meissner’s corpuscles is not stable throughout life, and its number and size are reduced with aging (McCormick et al., 2015). By 23 WEGA, axons with different morphologies are identified in the dermal papillae and occasionally penetrating the epidermis. However, for 10 WEGA, the axons remain “naked” since the S100 protein + precursors of the glial terminal cells, of rounded morphology, do not reach the dermal papillae until 33 WEGA. By 36–40 weeks S100 protein + cells also become vimentin + and progressively flattened. However, the typical morphology and arrangement of the lamellar cells of digital Meissner corpuscles are not observed until around 8 months of postnatal life (Feito et al., 2018).

### Pacinian Corpuscles

Pacinian corpuscles start development and structural differentiation in human digital skin by 13WEGA. At this time, and until 16 WEGA, the S100 protein + cells are organized in one or two layers of rounded cells, which progressively become smaller and flattened without evidence of lamellar organization (16 to 18 WEGA). These cells are the primigenial inner core. In parallel, the cells of the dermal surrounding mesenchyme organize to form the outer core and the capsule. Thereafter, between 20 and 24 WEGA, the S100 protein + cells emitted expansions that form a network and penetrate the outermost zone of the corpuscle, which later retract toward the central part of the corpuscle. Simultaneously, and extending between 24 and 36 WEGA, the edge of the inner core acquires a laminar arrangement, the lamellae becoming strongly packed and the inner core clefts being clearly distinguished. Nevertheless, the full maturation of Pacinian corpuscles in both morphology structure and expression of specific cell antigens is not totally defined until the fourth month of life. During the development of Pacinian corpuscles, expression of vimentin started shortly later than that of S100P and did not vary along the lifespan (Feito et al., 2018). In addition, Pacinian corpuscles generally showed no relevant age-related alterations (García-Piqueras et al., 2019).

On the other hand, Albuerne et al. (2000) analyzed the development of Meissner-like and Pacinian corpuscles in murine glabrous skin from the forepaw and hindpaw, as well as from the group of Pacinian corpuscles present in the interosseus membrane of the hindlimb. Although both kinds of sensory corpuscles start to develop prenatally, they become mature around postnatal day (Pd) 19–Pd28 for the Meissner-like corpuscles, and by Pd20 for the Pacinian ones. The lamellar cells in Meissner-like corpuscles expressed first the S100 protein (Pd7), then vimentin IR (Pd12), and transitory p75*^LNGFR^* (Pd7 to Pd19–20). In the Pacinian corpuscles, the lamellar cells forming the inner core displayed S100 protein by Pd7 and vimentin by Pd19 but lack p75*^LNGFR^*.

## Protein Profile of Glial Cells of Sensory Corpuscles

Cutaneous end-organ complexes have a very complex and heterogeneous protein content, as demonstrated in numerous immunohistochemical studies performed for more than 40 years. The references for papers reporting each of those proteins in the lamellar cells are included in the reviews by Vega’s lab (Vega et al., 1996, 2009; Cobo et al., 2021), Johansson’s lab (Johansson et al., 1999), and Pawson’s lab (Pawson et al., 2000).

Different cytoskeletal or cytosolic proteins have been detected in lamellar cells of sensory corpuscles. Some of them could be regarded as specific to those cells, but most of them are present in other cells where presumably they fulfill identical or similar functions. The intermediate filament protein filling the cytoplasm of the lamellar cells is vimentin instead of glial fibrillary acidic protein. However, differences seem to occur between species or anatomical localization since Pacinian corpuscles from human and cat peritoneum contain glial fibrillary acidic protein in addition to vimentin. Nevertheless, the coexpression of both cytoskeletal proteins was never observed; it is absent in human cutaneous sensory corpuscles. Interestingly, a subpopulation of lamellar cells in human digital Meissner and Pacinian corpuscles also display immunoreactivity for nestin, even in the adult life.

Several calcium-binding proteins, which presumably participate in the Ca^2+^ homeostasis and mechanoreceptor electrogenesis, are also detectable in the terminal glial cells of sensory corpuscles. They include S-100β, calbindin D28, calretinin, parvalbumin, and neurocalcin.

Also, some growth factors belonging to the family of the neurotrophins (BDNF) and their cognate receptors (p75*^NTR^*, TrkA, and TrkB), and epidermal growth factor receptor as well, are expressed by the terminal glial cells of cutaneous sensory corpuscles.

On the other hand, filling in spaces between lamellar cells, there is a chemically complex extracellular matrix formed by both fibrillary proteins and glycosaminoglycans, some of them organized as the basal lamina. In Meissner corpuscles, heparan sulfate proteoglycans were colocalized with type IV collagen, thus, suggesting that they are a part of the basal membrane, whereas chondroitin sulfate was absent. Regarding Pacinian corpuscles, the inner core contains decorin, biglycan, lumican, fibromodulin, and osteoadherin; the intermediate layer displays immunoreactivity for chondroitin sulfate and osteoadherin; was detected in the outer core lamellae and capsule, in the intermediate layer, and the inner core of Pacinian corpuscles expressing decorin, biglycan, lumican, fibromodulin, and osteoadherin (Vega et al., 1995; Dubový and Bednárová, 1999; García-Piqueras et al., 2019a,b, 2020b).

## The Role of the Lamellar Cells in Mechanotransduction

The axons and terminal glial cells of CEOCs are associated at multiple subcellular points, which can be observed using electron microscopy, in the so-called neuron receptive endings (NREs). NREs are specialized subcellular structures on sensory cells or neurons that receive inputs from either the environment or other neurons (Singhvi and Shaham, 2019). The sensory organs, including sensory corpuscles, have NREs exquisitely tuned to the sensory modality it transduces. Probably, NREs in sensory axons of CEOCs, and maybe also in the lamellar cells, could be related to the membrane regions where ion channels concentrate (see later). Classically, it is accepted that a key function of many glial cell subtypes is to modulate the NRE ionic microenvironment; for the terminal glia in CEOCs, it can be suggested that there is a modulation of the extracellular levels of K^+^, Na^+^, and Cl^–^, and probably also Ca^2+^ ions [for a review, see (Singhvi and Shaham, 2019; Reed et al., 2021)].

In this section, we review the putative role of lamellar cells in mechanosensing, updating the occurrence of mechano-gated ion channels and their possible involvement in mechanotransduction, as well as the data supporting neurotransmission in sensory corpuscles.

### Mechano-Gated Ion Channels in the Lamellar Cells

Touch is not only required for detection of objects and discrimination of shape, size, and texture but also for vibration. For touch perception, the first step is the conversion of mechanical stimuli into electrical activity, i.e., the receptor potential, and it occurs in cutaneous CEOCs. At present, the characteristics of the action potential generated in each CEOC subtype are rather well known, but the molecular events of this for mechanoelectrical transduction have not been fully identified.

The genesis of the receptor potential in the CEOCs of LTMRs was accepted that it depended on the mechanical properties of the periaxonal cells, especially the fibroblast of the outer core and the capsule, together with the characteristics of the axon membrane. In this mechanical theory, all the periaxonal cells are included in the term “capsule,” although the inner core, the intermediate layer, and the outer core capsule are completely different in their origin and cell composition. Thus, according to this theory, the mechanotransduction was regarded as a physical mechanical process. However, the discovery that mechanical forces can gate some ion channels present in the membranes of cutaneous, the somatosensory neurons including CEOCs, opened a new conception about the mechanisms of mechanotransduction (Sharif-Naeini, 2015).

A recent research has identified proteins essential for mechanotransduction and some others that can be required for some events of the mechanotransduction. Most of these proteins are related to ion channels (Martinac and Poole, 2018; Douguet and Honoré, 2019; Jin et al., 2020; Kefauver et al., 2020). Both voltage-gated and voltage-independent ion channels have been proposed to initiate mechanotransduction, and all have been localized in Meissner and/or Pacinian corpuscles primarily in the membrane of the axon but also in the terminal glial cells. Importantly, it must be underlined that the effect of a mechanical force on a cell depends on the site of incidence with the membrane. Cell membranes contain “protein corrals” (lipid-protein spatial domains), and the incidence of mechanical forces inside any domain is different from that in the surrounding membrane. So, the mechanosensitive ion channels can be gated or not by inclusion or exclusion from a domain (Sukharev and Sachs, 2012). Furthermore, it remains to be studied whether mechanosensitive ion channels are grouped on or in the vicinity of NREs (Singhvi and Shaham, 2019).

One of voltage-dependent K^+^ channels detected in a subpopulation of LTMRs is KCNQ4 (Kv7.4), which is crucial for setting the velocity and frequency preference in both mice and humans (Heidenreich et al., 2011). Moreover, the α-subunit of type I and type II voltage-gated Na^+^ channels are present in the axon and the non-neural inner and outer lamellae of rabbit Pacinian corpuscles. Those localizations suggest that they are involved in both transduction and action potential generation making it available to the axon *via* transport from the lamellae (Pawson and Bolanowski, 2002). Nevertheless, in this study, it is not clear whether the occurrence of voltage-gated Na^+^ channels occur in the inner core, the outer core–capsule, or both.

**FIGURE 3:**
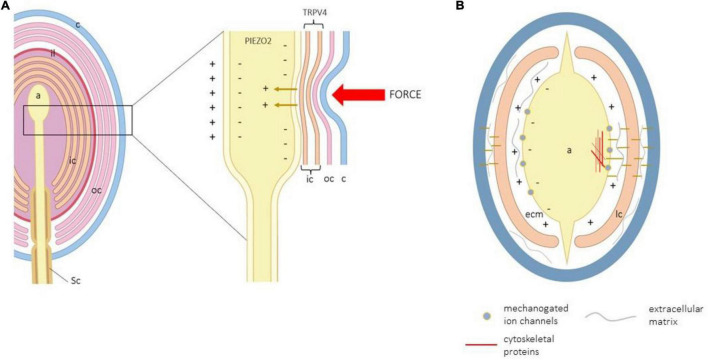
**(A)** Schematic representation of a Pacinian corpuscle showing the corpuscular structures that are modified by a force to generate the action potential. a, axon; c, capsule; ic, inner core; il, intermediate layer; lc, lamellar cells; oc, outer core; Sc, Schwann cells. **(B)** Schematic transversal section of a Pacinian corpuscle showing all cell structures potentially involved in mechanotransduction.

The voltage-independent mechano-gated ion channels fall into two main categories that respond to membrane tension or membrane stretch (Delmas and Coste, 2013). The opening of these channels allows the passage of ions into the axon to trigger the mechanotransduction (Gu and Gu, 2014; Ranade et al., 2015; Cobo et al., 2020). Thus, deformations in the membrane of the cells that form the mechanoreceptors open mechanosensitive ion channels that transduce mechanical energy into electrical activity. Therefore, if mechanotransduction starts in CEOCs, ion channels activated by force or displacement that act as mechanosensors and/or mechanotransducers should be expressed in the cells forming CEOCs. It should be clear that mechanical forces never directly reach the axon, which are ultimately responsible for triggering the potential for action. On the contrary, forces act on layers of cells, sometimes very numerous and of different natures, which in conjunction with the extracellular matrix and the cytoskeleton deform the axon membrane, opening the ion channels. In addition, the lamellae of terminal glial cells in CEOCs also express putative mechano-gated channels (Figure 3).

Channel complexes of the family of the degenerin/epithelial Na^+^ channels (DEG/ENa^+^C) might be involved in mechanotransduction. Ion channel subunits and family branches of DEG/ENa^+^C [like acid-sensing ion channels (ASIC)] are gated by mechanical forces and, for this reason, have been proposed as mechanotransducers [see for a review, (Ruan et al., 2021)]. DEG/ENa^+^C subunits and ASIC2 have been detected in the lamellar cells of murine and human Pacinian corpuscles (Montaño et al., 2009; Calavia et al., 2010) as well as in a subpopulation of human Meissner corpuscles (Cabo et al., 2015). ASIC2-knockout mice exhibit a decreased sensitivity of rapidly adapting cutaneous LTMRs, and disruption of ASIC3 reduces responses of cutaneous high-threshold mechanoreceptors to noxious stimuli (Drew et al., 2004). Nevertheless, the role of ASIC in cutaneous mechanosensitivity is doubted (Page et al., 2004).

Nearly all TRP (transient receptor potential) families of ion channels have members with potential mechanosensory capacities (Clapham et al., 2005; Samanta et al., 2018). Nevertheless, it has not been established whether these ion channels are mechanosensors or participate only when required for mechanosensation since some of them are insensitive to membrane stretch [see (Nikolaev et al., 2020)]. As far as we know, TRPV4 (vanilloid 4) is the only member of the TRP superfamily that has been detected in the lamellar cells of human digital Meissner (Alonso-González et al., 2016). Nevertheless, a role for this protein within the lamellar cells of sensory corpuscles remains to be demonstrated, although disruption of *trpv4* in mice leads to insensitivity to pressure sensation in the mouse tail (Suzuki et al., 2003).

The occurrence of the members of the other two families of ion channels involved in mechanotransduction, i.e., the family of two pore domain channels and Piezo have been never detected in the terminal glial cells of CEOCs. More recently, the proteins codified by the Piezo gene, PIEZO1 and PIEZO2, have proven to have true mechanosensory ability and are directly involved in mechanotransduction (Coste et al., 2010; Ranade et al., 2014). PIEZO2 function as a transducer in LTMRs and Merkel cells, such as the lamellar cells, can be regarded as sensing cells in Merkel cell–neurite complexes and are associated with the axon terminal of Aβ mechanosensory fibers. In the murine skin, PIEZO2 is expressed and has functional expression present in Merkel discs and isolated Merkel cells (Ikeda et al., 2014; Maksimovic et al., 2014; Woo et al., 2014, 2015a,b), Meissner-like corpuscles, and lanceolate nerve endings (Ikeda et al., 2014). PIEZO2 has been also detected in human Merkel cells and Meissner’s corpuscle axon, in an age-dependent manner (Sedý et al., 2004; García-Mesa et al., 2017). Consistently with those localizations, PIEZO2-deficient mice show an almost complete deficit in light-touch sensation and proprioception with preserved function in other somatosensory modalities (Ranade et al., 2014). PIEZO2 mutations in human patients lead to selective loss of touch perception and heavily decreased proprioception (Chesler et al., 2016; Mahmud et al., 2017).

The most overwhelming putative role of lamellar cells in mechanosensation was provided recently by Nikolaev et al. (2020). They observed that the lamellar cells of Grandry’s corpuscles (assimilated to the lamellar cells within Meissner corpuscles) from duck bill skin detect tactile stimuli, thus, suggesting that also lamellar cells are touch sensors. The authors observed that Grandry’s cells act as non-neuronal mechanosensors that contain mechanically gated ion channels and can generate robust Ca^2+^-dependent action potentials *via* R-type Cav channels. Furthermore, they also showed that outer-core lamellar cells from Herbtst corpuscles (equivalent to the mammalian Pacinian corpuscles) are mechanosensitive, but unlike lamellar cells in Grandry’s corpuscles, they are not excitable. Consistently, these propose that Meissner and Pacinian corpuscles use neuronal and non-neuronal mechanoreception to detect mechanical signals. It may be worth to note that even the structures are not identical; there is striking similarity between microanatomy, function, and electrophysiology of the avian Grandry’s and Herbtst corpuscles vs. Meissner and Pacinian corpuscles, respectively.

The extracellular matrix and cytoskeletal proteins anchored to the extra- or intracytoplasmic domains, respectively, of the cell membrane in the vicinity of mechano-gated ion channels could play important roles in mechanotransduction. As mentioned in previous paragraphs, the force exerted on the skin does not act directly on the axon that generates the action potential. Among the skin surface and the axon membrane are the different layers of the epidermis, the epidermal–dermic connections, and the transmission from the dermis through a greater or lesser number of cell layers (capsule and laminar cells in the Meissner corpuscles; capsule, outer core, intermediate lamina, and inner core in the Pacini corpuscles) until the axon. Therefore, we consider that the role of the extracellular matrix, the cytoskeletal proteins, and the cell-to-cell junctions in CEOCs are of capital importance in the process of mechanosensing. Integrins (Dubovy et al., 1999) and other linking extracellular matrix proteins present in sensory corpuscles could participate in mechanosensing and/or mechanotransduction. For example, integrin α1β1 is necessary for the function of TRPV4 ion channel in chondrocytes (Jablonski et al., 2014) and influence the density of Meissner-like corpuscles in murine footpads (Wai et al., 2021). Recently, Schwaller et al. (2021) found in the lamellar cells of Meissner-like corpuscles USH2A, a transmembrane protein with a very large extracellular domain, and in the absence of this protein, the RA type I LTMRs showed large reductions in vibration sensitivity.

### Putative Synaptic Mechanism in the Lamellar Cells

In Pawson et al. (2007, 2009) published a series of elegant studies that suggested that in cats, Pacinian corpuscle classical neurotransmission cannot be excluded for the genesis of the action potential, involving both the axons and the periaxonal cells. This is in addition to the opening of voltage-gated and non-voltage-gated channels as responsible, or at least necessary, for mechanotransduction. The authors specifically speak about the lamellar cells of the terminal glial but only of periaxonal cells (“*modified Schwann cells of the inner core*”).

**FIGURE 4:**
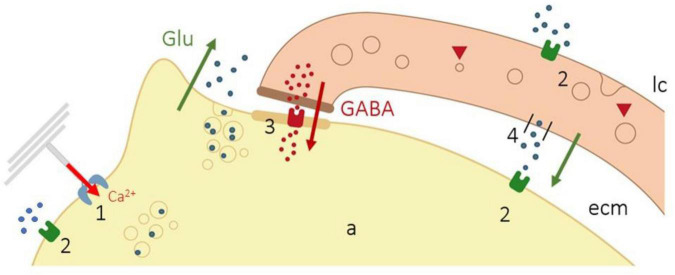
Schematic representation of the synaptic-like coupling of the axon and lamellar cells in Pacinian corpuscles. Opening of mechano-gated channels (1) causes the entry of Ca^2+^ in the axon (red arrow) and release of glutamate from clear-core vesicles of the axon (green arrow), which can act on the lamellar cells or the axon (2). Glutamate acting on lamellar cells induces GABA release (brown arrow), which activates GABA receptors in the axon (3) inhibiting glutamate excitation. In addition, the lamellar cells contain vesicular-glutamate transporters, and SNARE proteins, that consent glutamate release by the lamellar cells (4). Modified from Pawson et al. (2009).

The studies of Pawson and coworkers postulated that “action potentials in response to dynamic stimuli are due to depolarization of the axon by cations entering mechano-gated channels that are opened due to mechanical motion; however, action potentials in the static portion of the Pacinian corpuscle rapidly adapting response are due to glutamatergic excitation, which are then inhibited by GABA released from the modified Schwann cells of the inner core.” This synaptic-like activity is based on the following observations: (a) lamellar cells contains the machinery to synthesize, store (express immunoreactivity for the synaptic proteins synaptobrevin VAMP2 and SNAP-23), and release neurotransmitters (glutamate, GABA); (b) lamellar cells release neurotransmitters when they are stimulated by glutamate, ATP, or even by mechanical motion; and (c) the lamellar cells express glutamate receptors. Thus, the sequence for mechanotransduction based on the synaptic-like theory is as follows: After initial opening of mechano-gated channels by mechanical stimulus, the entry of Ca^2+^ in the axon and the subsequent depolarization that originates the receptor potential induce the release of glutamate from clear-core vesicles of the axon. Glutamate can act either on the lamellar cells, the axon, or both the lamellar cells and the axon, which express glutamate receptors. Glutamate acting on lamellar cells induces GABA release, which, acting on GABA-receptors of the axon, inhibits glutamate excitation. In addition, the lamellar cells contain vesicular-glutamate transporters, and SNARE proteins, that consent glutamate release by the lamellar cells. Importantly, the mechanical stimulus alone may be responsible for the release of either the glutamate or the GABA from the lamellar cells (Figure 4).

These findings argue for a chemical, possibly bidirectional, interaction between the lamellar cells and the axon. This “mechanochemical” theory for the genesis of mechanotransduction has been proposed only in Pacinian corpuscles and remains to be demonstrated that similar mechanisms of neurotransmission occur in other kinds of sensory corpuscles. Nevertheless, it is well documented that mechanosensory axon terminals, but not the associated terminal glial cells, have “an apparently ubiquitous glutamate secretory system” of unknown functional significance [see for review (Bewick, 2015)]. The only cells associated with Aβ afferents are Merkel cells that establish synapsis-like contact at Merkel cell–neurite complexes. Consistently, Merkel cells, regarded as the presynaptic cells, contain all the components of the presynaptic machinery (active-zone molecules and synaptic vesicle proteins) and release neurotransmitters (Haeberle et al., 2004; Maksimovic et al., 2013; Nakatani et al., 2015).

## Concluding Remarks

The terminal glial cells of CEOCs are a subpopulation of peripheral glial cells, highly differentiated, with putative roles in mechanosensing and/or mechanotransduction. In the last decade, some mechano-gated ion channels have been discovered in terminal glial cells. This fact, associated with the presence of some components of a GABA-ergic/glutamatergic neurotransmission system in the Pacinian corpuscles, suggests that the glial cells of sensory corpuscles are not passive supporting cells but could have an active role in the mechanotransduction process and the genesis of the action potential.

## Data Availability Statement

The raw data supporting the conclusions of this article will be made available by the authors, without undue reservation.

## Author Contributions

All authors listed have made a substantial, direct, and intellectual contribution to the work, and approved it for publication.

## Conflict of Interest

The authors declare that the research was conducted in the absence of any commercial or financial relationships that could be construed as a potential conflict of interest.

## Publisher’s Note

All claims expressed in this article are solely those of the authors and do not necessarily represent those of their affiliated organizations, or those of the publisher, the editors and the reviewers. Any product that may be evaluated in this article, or claim that may be made by its manufacturer, is not guaranteed or endorsed by the publisher.
